# Impact of age on pneumococcal colonization of the nasopharynx and oral cavity: an ecological perspective

**DOI:** 10.1093/ismeco/ycae002

**Published:** 2024-01-12

**Authors:** Willem R Miellet, Rob Mariman, Janieke van Veldhuizen, Paul Badoux, Alienke J Wijmenga-Monsuur, David Litt, Thijs Bosch, Elizabeth Miller, Norman K Fry, Marianne A van Houten, Nynke Y Rots, Elisabeth A M Sanders, Krzysztof Trzciński

**Affiliations:** Department of Pediatric Immunology and Infectious Diseases, University Medical Center Utrecht (UMCU), Wilhelmina Children's Hospital, Utrecht, 3584 CX, The Netherlands; Centre for Infectious Disease Control Netherlands, National Institute for Public Health and the Environment (RIVM), Bilthoven, 3721 MA, The Netherlands; Centre for Infectious Disease Control Netherlands, National Institute for Public Health and the Environment (RIVM), Bilthoven, 3721 MA, The Netherlands; Centre for Infectious Disease Control Netherlands, National Institute for Public Health and the Environment (RIVM), Bilthoven, 3721 MA, The Netherlands; Regional Laboratory of Public Health (Streeklab) Haarlem, Haarlem, 2035 RC, The Netherlands; Centre for Infectious Disease Control Netherlands, National Institute for Public Health and the Environment (RIVM), Bilthoven, 3721 MA, The Netherlands; Respiratory and Vaccine Preventable Bacterial Reference Unit (RVPBRU) and Immunisation and Vaccine Preventable Diseases Division, UK Health Security Agency, London, NW9 5EQ, United Kingdom; Centre for Infectious Disease Control Netherlands, National Institute for Public Health and the Environment (RIVM), Bilthoven, 3721 MA, The Netherlands; School of Hygiene and Tropical Medicine, Department of Infectious Disease Epidemiology, London, WC1E 7HT, United Kingdom; Respiratory and Vaccine Preventable Bacterial Reference Unit (RVPBRU) and Immunisation and Vaccine Preventable Diseases Division, UK Health Security Agency, London, NW9 5EQ, United Kingdom; Department of Pediatrics, Spaarne Gasthuis, Haarlem, 2035 RC, The Netherlands; Centre for Infectious Disease Control Netherlands, National Institute for Public Health and the Environment (RIVM), Bilthoven, 3721 MA, The Netherlands; Department of Pediatric Immunology and Infectious Diseases, University Medical Center Utrecht (UMCU), Wilhelmina Children's Hospital, Utrecht, 3584 CX, The Netherlands; Centre for Infectious Disease Control Netherlands, National Institute for Public Health and the Environment (RIVM), Bilthoven, 3721 MA, The Netherlands; Department of Pediatric Immunology and Infectious Diseases, University Medical Center Utrecht (UMCU), Wilhelmina Children's Hospital, Utrecht, 3584 CX, The Netherlands

**Keywords:** Streptococcus pneumoniae, serotype, carriage, saliva, nasopharynx, dissimilarity, age

## Abstract

Pneumococcal carriage studies have suggested that pneumococcal colonization in adults is largely limited to the oral cavity and oropharynx. In this study, we used total abundance-based β-diversity (dissimilarity) and β-diversity components to characterize age-related differences in pneumococcal serotype composition of respiratory samples. quantitative PCR (qPCR) was applied to detect pneumococcal serotypes in nasopharyngeal samples collected from 946 toddlers and 602 adults, saliva samples collected from a subset of 653 toddlers, and saliva and oropharyngeal samples collected from a subset of 318 adults. Bacterial culture rates from nasopharyngeal samples were used to characterize age-related differences in rates of colonizing bacteria.

Dissimilarity in pneumococcal serotype composition was low among saliva and nasopharyngeal samples from children. In contrast, respiratory samples from adults exhibited high serotype dissimilarity, which predominantly consisted of abundance gradients and was associated with reduced nasopharyngeal colonization. Age-related serotype dissimilarity was high among nasopharyngeal samples and relatively low for saliva samples.

Reduced nasopharyngeal colonization by pneumococcal serotypes coincided with significantly reduced *Moraxella catarrhalis* and *Haemophilus influenzae* and increased *Staphylococcus aureus* nasopharyngeal colonization rates among adults.

Findings from this study suggest that within-host environmental conditions, utilized in the upper airways by pneumococcus and other bacteria, undergo age-related changes. It may result in a host-driven ecological succession of bacterial species colonizing the nasopharynx and lead to competitive exclusion of pneumococcus from the nasopharynx but not from the oral habitat. This explains the poor performance of nasopharyngeal samples for pneumococcal carriage among adults and indicates that in adults saliva more accurately represents the epidemiology of pneumococcal carriage than nasopharyngeal samples.

## Introduction

*Streptococcus pneumoniae* (pneumococcus) is an important human pathogen and a common colonizer of the respiratory tract. Within the upper airways, pneumococcus competes with other bacteria for limiting resources, which are often derived from the host. Competitive dominance for limiting resources of the respiratory tract is likely an important determinant of pneumococcal colonization [1]. In ecology, such consumer-resource dynamics of species are recognized as major drivers of interspecies competition and community composition and can be used to define the ecological niche of a species [2, 3]. According to consumer-resource theory, resource availability in a local environment and resource utilization trade-offs can dictate whether a species is likely to become competitively dominant [2–6]. To this end, shifts in host-derived resources with age may impact whether pneumococcus can successfully colonize the respiratory tract of a human host.

In young children, nasopharyngeal colonization by *S. pneumoniae* is commonly observed and these young carriers typically display high absolute and relative pneumococcal abundances in the nasopharynx [7]. In comparison, pneumococcal colonization of the nasopharynx is relatively rare among adults, and pneumococcal abundances in this age group are usually low. Moreover, findings from numerous pneumococcal carriage studies, including studies employing molecular-based methods, have indicated that pneumococcal colonization among adults is more frequently observed in oral samples than in nasopharyngeal samples [8–11].

Remarkably, age-related declines in pneumococcal colonization rates of the nasopharynx coincide with other shifts in nasopharyngeal microbiota composition. Stearns *et al*. compared the microbiota of the oropharynx and nasopharynx between children and adults. In this study, oropharyngeal samples exhibited few age-related differences in microbiota composition, while nasopharyngeal samples displayed strong age-related declines in *Pseudomonadota*, in particular *Moraxella*, and a rise in *Bacillota*, notably *Staphylococcus* [12]. Matching age-related differences in the microbiota composition of the nasopharynx have also been documented by others [13, 14].

In this study, we characterized the variation in pneumococcal serotype composition of respiratory samples from community-dwelling individuals consisting of children under 5 years of age and their parents, and proposed a conceptual ecological model of age-related differences in carriage. We observed age-related dissimilarity in the pneumococcal serotype composition among nasopharyngeal and saliva samples from children and adults. These findings suggest that the environmental conditions of the nasopharynx in young children are likely to be transient and subject to age-related differences. Such age-related changes of the nasopharynx can result in host-driven ecological succession of bacterial species colonizing the nasopharynx and may explain poor performance of nasopharyngeal samples for surveillance of pneumococcal carriage among adults. Our observations suggest that pneumococci detected using saliva samples represent more accurately the epidemiology of pneumococcal carriage among adults.

## Materials and methods

### Study design and ethics statement

Pneumococcal carriage was studied in cross-sectional prospective observational studies conducted in 2015/2016 in the Netherlands [15] and in England [16]. Approval for the Dutch study was given by the Medical Ethics Committee Noord Holland and is registered (reference NTR5405 on https://trialsearch.who.int/), and for the English study by the NHS Health Research Authority and the London Fulham Research Ethics Committee (reference 15/LO/0458) and is registered (reference NCT02522546 on https://trialsearch.who.int/). For every participating child, written informed consent was obtained from the parents or guardian, and adults produced written consent for their own participation. The studies were conducted in accordance with Good Clinical Practice and the Declaration of Helsinki.

### Sample collection and laboratory processing

In the Netherlands, nasopharyngeal and saliva samples were collected from *n* = 653 children vaccinated with 10 valent pneumococcal conjugated vaccine (PHiD-CV, GlaxoSmithKline), including *n* = 327 24-month-old vaccinated with PHiD-CV according to “2 primary + 1 booster” dose schedule and *n* = 326 children aged 44–49 months (“4-year-old”) vaccinated following a “3 primary + 1 booster” schedule. In addition, nasopharyngeal, oropharyngeal, and saliva samples were collected from *n* = 318 parents of 24-month-old (“2-year-old”, one parent per child). In England, nasopharyngeal swabs were collected from *n* = 293 children aged 1–5 years vaccinated with 13-valent conjugated polysaccharide vaccine (Prevnar-13, Pfizer) in a “2 primary +1 booster” dose schedule and from *n* = 284 adult household contacts of the children [16].

Nasopharyngeal and oropharyngeal swabs were collected and processed in accordance with the protocol recommended by the World Health Organization [17]. Procedures for collection of samples in The Netherlands [7, 9, 15] and in England [16] are detailed in supplementary materials.

### Detection of respiratory bacteria using conventional diagnostic culture

All nasopharyngeal swabs collected in the Netherlands were cultured to determine the presence of *S. pneumoniae*, *Staphylococcus aureus*, *Haemophilus influenzae*, and *Moraxella catarrhalis* using standard bacteriological procedures for conventional culture. In England, nasopharyngeal samples were cultured exclusively for *S. pneumoniae*. In both studies, one pneumococcal colony per sample was subcultured and serotyped in the Netherlands by Quellung reaction using type-specific antisera from the Statens Serum Institute (Copenhagen, Denmark) [15]. In England, serotyping was conducted through whole-genome sequencing of pneumococcal isolates and serotype prediction using the PneumoCaT bioinformatic pipeline, supplemented with slide agglutination when required [16, 18].

### Molecular detection of *S. pneumoniae* and pneumococcal serotypes

Detection of *S. pneumoniae* and pneumococcal serotypes in nasopharyngeal, oropharyngeal, and saliva samples has been detailed previously [7, 9]. In short, respiratory samples were cultured using SB7-Gent agar (Oxoid, Badhoevedorp, The Netherlands) plates to enrich samples for the potential presence of pneumococcus. Both raw (minimally processed) and culture-enriched samples were then processed for DNA extraction. Pneumococcal DNA was quantified using a “Two-to-Tango” (or dual-target) approach with singleplex qPCRs specific for DNA sequences within genes encoding for pneumococcal iron uptake ABC transporter lipoprotein PiaB [10] and for major pneumococcal autolysin LytA [19]. A sample was considered positive for *S. pneumoniae* by qPCR when C_q_s for both targeted genes were below thresholds determined for a particular sample type with receiver operating characteristic (ROC) curve analysis using amplification slopes as criterion [9].

Next, DNA extracts from culture-enriched samples were used to determine serotype composition of respiratory samples with singleplex qPCR assays. We used 29 sets of primers and probes [20–22] targeting 53 serotypes including the 24 vaccine serotypes covered by those pneumococcal vaccines available in the Netherlands or in England, namely PHiD-CV (GlaxoSmithKline), PCV13 (Pfizer), and 23-valent polysaccharide vaccine PPV23 (Merck Sharp & Dohme). The panel also targeted a selection of non-vaccine serotypes, namely serotypes 6C, 6D, 7A, 9A, 9L, 10B, 11D, 12A, 12B, 15A, 15C, 15F, 18A, 18B, 18F, 16F, 21, 22A, 22F, 23A, 23B, 33A, 34, 35B, 35C, 37, and 38. With several qPCR assays, it was not possible to distinguish between serotypes within a serogroup, specifically 6A and 6B; 6C and 6D; 7A and 7F; 9A, 9L, 9N and 9V; 10A and 10B; 11A and 11D; 12A, 12B and 12F; 15A, 15B, 15C and 15F; 22A and 22F; 33A, 33F and 37; 35B and 35C. Primers and probes used in these assays and their concentrations were described previously [7]. A sample was considered positive for a certain pneumococcal serotype when the sample was previously classified as positive for *S. pneumoniae* by qPCR and when serotype-specific qPCR measurements were equal or below specimen-specific *piaB* C_q_ thresholds [9].

### 16S quantification by qPCR

Overall bacterial abundances in all minimally processed nasopharyngeal, oropharyngeal, and saliva samples were measured using 16S rRNA gene-targeted qPCR (Fw primer: 5′-CGAAAGCGTGGGGAGCAAA-3′, Rv primer: (5′-GGTCGTACTCCCCAGGCGG-3′, probe: 5′-FAM-ATTAGATACCCTGGTAGTCCA-3′-TAMRA) [23] and were used to compare overall bacterial abundances [24].

### Data analysis

Data analysis was performed in R version 4.3.2. We used “amplification slopes” as a criterion to compute C_q_ thresholds with ROC curve analysis [9]. Here, we regarded a sample as negative when it exhibited no decrease in both *piaB* and *lytA* C_q_s after culture-enrichment when compared with the corresponding paired minimally processed sample. Of note, C_q_s are inversely proportional to the log of the template DNA concentration. The specimen-specific (and cohort-specific) C_q_ thresholds (Table S1) were then used to classify qPCR-based serotyping results as positive or negative. Frequencies in paired comparisons were tested using the McNemar’s test, and unpaired comparisons were tested using Fisher’s Exact test. Comparisons between abundances quantified with qPCR were tested with Mann–Whitney U test. Frequencies of pneumococcal serotypes (detected with qPCR exclusively) and cultured bacterial species were used as a substitute for species abundance to estimate dissimilarity between specimen types, age groups, or combinations of these, where serotype-specific carriage events were considered as a single unit. This approach to dissimilarity estimation in ecological studies has been described elsewhere [25]. Abundances were square-root transformed to limit the effect of highly prevalent serotypes [26]. We used rarefaction to control for variation in sampling of each study group and evaluated sampling coverage with the “íNext” R package [27]. In this study, the study group with the smallest size consisted of data from 284 individuals. Consequently, we randomly sampled 284 individuals 100 times for each specimen-type-age-group combination (equivalent of 100 carriage studies). Abundance-based β-diversity (Bray–Curtis index), interpreted as site-to-site dissimilarity, was from groups of samples. Total dissimilarities between samples were partitioned into abundance gradients (analogous to nestedness) and balanced variation (analogous to turnover) using the “betapart” R package [28]. We performed non-metric multidimensional scaling (NMDS) to plot results from a Bray–Curtis dissimilarity matrix. We used a probabilistic model of co-occurrence with the “cooccur” R package to identify positive and negative associations among cultured bacterial species, probabilities below 0.05 were considered significant [29]. Consumer-resource models of switching resource type were made with the Populus software (D.N. Alstad, University of Minnesota, v5.5). A *P*-value of <.05 was regarded as significant.

## Results

Upper respiratory tract (URT) samples from 946 children aged 1–5 years (*n* = 653 from the Netherlands and *n* = 293 from England) and 602 adults (*n* = 318 from the Netherlands and *n* = 284 from England) were characterized with qPCR-based detection for the composition of pneumococcal serotypes.

A total of 814 serotype carriage events were detected from 2837 respiratory samples (Tables S2–S6). Serotype-specific qPCR assays targeting 4, 5, 9A/9L, 12A/12B/12F, and 35B/35C were excluded from analysis because Bland–Altman analysis indicated poor concordance for these particular assays between *piaB/lytA* C_q_s and serotype-specific C_q_s in one or more type of sample investigated [9]. Pneumococcal serotype composition was compared for each age-group-specimen-type combination. Coverage-based rarefaction analysis indicated that all age groups were sufficiently sampled to characterize the pneumococcal serotype composition of individuals (Fig. S1). Oropharyngeal and saliva samples from adults displayed high sampling coverage, whereas nasopharyngeal samples from adults exhibited insufficient sampling coverage. However, we attribute this to a biological effect rather than to insufficient sampling of adults, as overall results per age group indicated sufficient sampling coverage.

In a comparison of nasopharyngeal samples from England and the Netherlands, we noted substantial age-related differences in serotype composition using abundance-based dissimilarity (Bray–Curtis index: >0.60) within both cohorts (Table 1), suggesting limited similarity in serotype composition of nasopharyngeal samples between age groups. Age-related serotype dissimilarity was predominantly composed of abundance gradients (analogous to nestedness), indicating that although serotypes detected in adults were of the same types as those detected in children, overall serotype-specific prevalence was substantially reduced in nasopharyngeal samples from adults. Between nasopharyngeal samples from children in the Netherlands and England, low to moderate serotype dissimilarity (<0.30) was observed, which predominantly consisted of balanced variation (analogous to turnover). This indicated that certain pneumococcal serotypes were more prevalent in England when compared with the Netherlands and *vice versa*. Indeed, serogroup 6A/6B/6C/6D and serotype 19A, vaccine serotypes included in the PCV13 vaccine but not the PHiD-CV vaccine, were more common in children from the Netherlands, whereas serogroup 15A/15B/15C/15F and serotype 21 were more prevalent in children from England compared with 4-year-old children from the Netherlands.

**Table 1:** Serotype dissimilarity among nasopharyngeal samples collected from individuals in the Netherlands (NL) and in England (ENG).

Comparison	Serotype dissimilarity	Abundance gradients (β ratio)	Balanced variation (β ratio)
NL 2-year-old children vs NL 4-year-old children	0.16	0 (0.02)	0.15 (0.98)
NL 2-year-old children vs ENG 0–4-year-old children	0.21	0.04 (0.21)	0.17 (0.79)
NL 4-year-old children vs ENG 0–4-year-old children	0.26	0.04 (0.15)	0.22 (0.85)
NL 2-year-old children vs NL adults	0.65	0.65 (1)	0 (0)
NL 4-year-old children vs NL adults	0.69	0.58 (0.85)	0.11 (0.15)
ENG 0–4-year-old children vs NL adults	0.66	0.56 (0.84)	0.11 (0.16)
NL 2-year-old children vs ENG adults	0.87	0.87 (1)	0 (0)
NL 4-year-old children vs ENG adults	0.87	0.87 (1)	0 (0)
ENG 0–4-year-old children vs ENG adults	0.86	0.86 (1)	0 (0)
NL adults vs ENG adults	0.68	0.33 (0.49)	0.35 (0.51)

Mean serotype dissimilarity was calculated after rarefaction and by using a resampling procedure with *n* = 100 times for each group, NL: cohort from the Netherlands, ENG: cohort from England.

**Figure 1:**
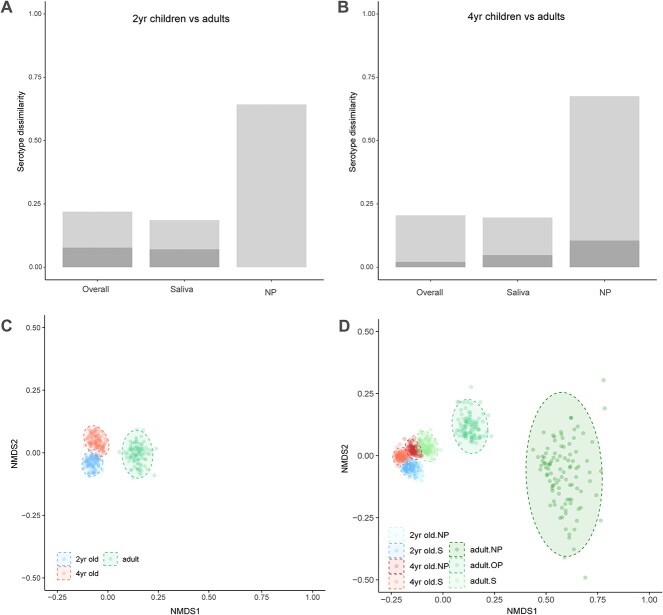
Differences in serotype composition between age groups and specimen-type-age-group combinations; (A) mean serotype dissimilarity (Bray–Curtis index) among nasopharyngeal and saliva samples from 2-year-old children and adults, and overall dissimilarity (either nasopharyngeal or saliva); (B) mean serotype dissimilarity 4-year-old children and adults; overall serotype dissimilarity and serotype dissimilarity among saliva samples was low (<0.25), while serotype dissimilarity among nasopharyngeal samples was high (>0.50) and consisted primarily of abundance gradients (light grey) and not balanced variation (dark grey); (C) NMDS of overall serotype dissimilarity calculated from serotype occurrence data from nasopharyngeal and saliva samples and (D) of each specimen-type-age-group combination; serotype dissimilarity was calculated after rarefaction and by means of a resampling procedure with *n* = 100 times for each group. Stress for C and D was 11.3% and 6.8%, respectively; ellipses indicate 95% confidence intervals of normal distribution of Bray–Curtis index for each group.

Next, we used the study cohort from the Netherlands to characterize the pneumococcal serotype composition of saliva and nasopharyngeal samples in children, while in case of adults we also included oropharyngeal samples. When considering serotype rates from both saliva and nasopharyngeal samples together, overall dissimilarity was low between children and adults (Fig. 1). The number of detected pneumococcal serotypes was higher in saliva as compared with nasopharyngeal samples, also among samples from children (Table S7). The diversity of pneumococcal serotypes detected in adult saliva samples resembled that of nasopharyngeal and saliva samples from children (Shannon diversity 2.59 of adult saliva compared to 2.37–2.60 of nasopharyngeal and saliva samples from children). Nasopharyngeal and saliva samples from children were similar in serotype composition as serotype dissimilarity was low (<0.15). Also, saliva samples from adults exhibited little age-related dissimilarity in serotype composition when compared to nasopharyngeal or saliva samples from children, but nasopharyngeal samples showed substantial dissimilarity (Fig. 1). A comparison of URT samples from adults also indicated that age-related differences in serotype composition were largely limited to nasopharyngeal samples since substantial serotype dissimilarity due to abundance gradients was observed between both saliva and nasopharyngeal samples and oropharyngeal and nasopharyngeal samples. Saliva and oropharyngeal samples from adults exhibited low serotype dissimilarity when comparing age groups. Age-related differences in nasopharyngeal serotype composition coincided with declines in the abundances of pneumococci (Fig. S2A and B).

**Figure 2:**
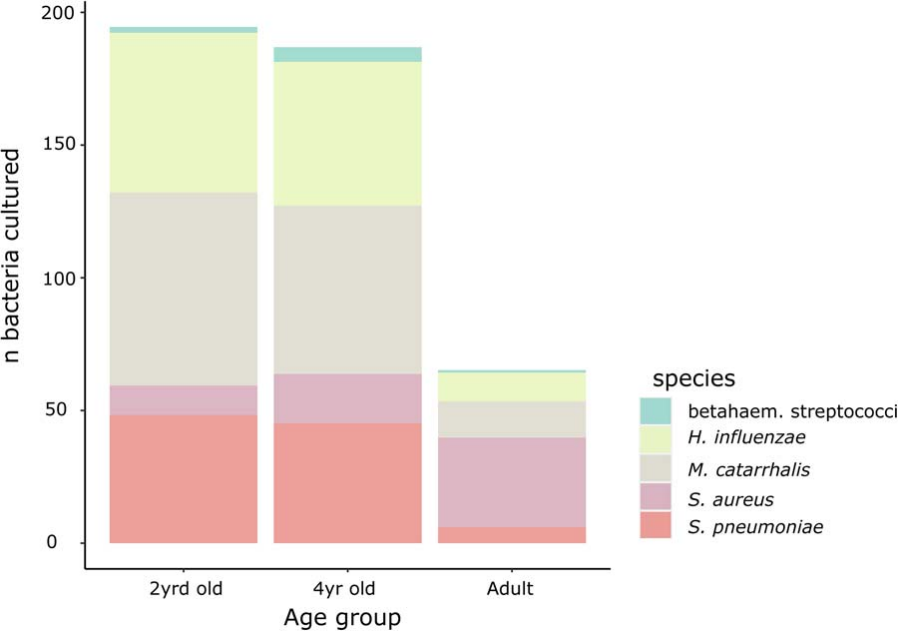
Distribution of bacterial species cultured from nasopharyngeal samples collected in the Netherlands from 2-year-old children (*n* = 327), 4-year-old children (*n* = 326), and parents of 2-year-old children (*n* = 318); of note: *M. catarrhalis* is significantly less prevalent (Fisher’s exact test, *P* = .01) and *S. aureus* is more prevalent (Fisher’s exact test, *P =* .008) among 4-year-old compared with 2-year-old; all comparisons (except for β-haemolytic streptococci; *S. pyogenes, S. dysgalactiae, S. anginosus*) between children (for 2-year-old and 4-year-old separately) and adults are significant with *S. pneumoniae, H. influenzae*, and *M. catarrhalis* (Fisher’s exact test, *P* < .0001 for all) displaying reduced and *S. aureus* (Fisher’s exact test, *P <* .0001) displaying increased prevalence rates in adults.

Thereafter, we assessed whether differences among nasopharyngeal samples, presumed to be due to age-related effects, also impacted colonization by other common bacterial species. To this end, we compared nasopharyngeal culture rates (Table S8) and overall bacterial abundances (based on 16S rRNA gene-targeted qPCR) between age groups in the study cohort from the Netherlands. Among children aged 2 and 4 years, nasopharyngeal colonization by *S. pneumoniae* was positively correlated with the presence of *H. influenzae* and *M. catarrhalis*, yet negatively correlated with *S. aureus* (Tables S9 and S10). Likewise, nasopharyngeal colonization by *H. influenzae* was positively correlated with *M. catarrhalis* and both were negatively correlated with *S. aureus*. Adults displayed diminished nasopharyngeal colonization rates for pneumococcus, *M. catarrhalis*, and *H. influenzae* when compared with children, and only *H. influenzae* was positively correlated with *M. catarrhalis* (Fig. 2, Table S11). In contrast, nasopharyngeal colonization by *S. aureus* was significantly increased in adults.

Finally, overall bacterial abundances in both nasopharyngeal and saliva samples declined with age (Fig. S2C and D). It coincided with the observed age-related dissimilarity of colonizing pneumococcal serotypes. Nasopharyngeal samples positive for either *S. pneumoniae*, *H. influenzae*, or *M. catarrhalis* were characterized by higher overall bacterial abundances when compared with samples culture negative for the corresponding bacterial species (Fig. S3A–C). Conversely, nasopharyngeal samples positive for *S. aureus* by culture displayed reduced overall bacterial abundances when compared with samples negative by culture for *S. aureus* (Fig. S3D).

## Discussion

In this study, we have characterized the pneumococcal serotype composition of URT samples collected from children under 5 years of age and adults. We observed substantial age-related serotype dissimilarity among nasopharyngeal samples. Age-related serotype dissimilarity was predominantly composed of the abundance gradient component of β-diversity and was due to overall declines in nasopharyngeal colonization rates by pneumococcus in adults. Adult pneumococcal carriers who did exhibit nasopharyngeal colonization displayed diminished pneumococcal abundances when compared with children. These findings are in line with studies from our group and others, already describing low sensitivity of nasopharyngeal swabs in detection of pneumococcal carriage in adults [10, 11, 30, 31]. Prior to these contemporary observations, early investigators during the 20th century already noted that adult pneumococcal carriers were often found to lack nasopharyngeal colonization, while pneumococcus was cultured instead from the oral cavity (e.g. positive in saliva) or oropharynx [8, 32–34]. Collectively, the findings from this study and other studies suggest that age-related changes may occur in the nasopharynx, which may limit pneumococcal colonization of the nasopharynx in adulthood.

A comparison between children and adults indicated that dissimilarity was lowest between these age groups when saliva was applied for serotype detection in adults. These observations are in agreement with studies describing enhanced sensitivity of pneumococcal detection with oral samples [10, 11] and it underlines that pneumococci detected in saliva may more accurately resemble the epidemiology of pneumococcal carriage among adults than those detected in nasopharyngeal samples. Saliva samples exhibited increased richness in pneumococcal serotypes when compared with nasopharyngeal samples. This observation corresponded to higher rates of co-carriage in saliva samples when compared with nasopharyngeal samples [9] and suggested that pneumococci in the oral cavity may have access to more resources, for example dietary carbohydrates such as inulin [35]. Moreover, since dissimilarity in serotype composition among nasopharyngeal and saliva samples was predominantly composed of abundance gradients and not balanced variation, it indicated a general reduction in serotype colonization in the nasopharynx. Accordingly, an unmasking effect of serotypes (e.g. vaccine-type serotypes) was not evident in oral samples. The number of detected serotypes in children was higher in saliva samples than in nasopharyngeal swabs, and dissimilarity in serotype composition between nasopharyngeal and saliva samples was primarily driven by abundance gradients. The higher number of detected serotypes in saliva samples corresponded to higher rates of co-carriage in saliva samples when compared to nasopharyngeal samples [9], suggesting that paediatric carriage studies limiting testing to nasopharyngeal samples may underestimate serotype-specific prevalence rates.

No marked differences in serotype composition of nasopharyngeal and saliva samples from children were found. However, a comparison of serotype-specific rates among nasopharyngeal samples from England and the Netherlands indicated higher rates of serogroup 6A/6B/6C/6D and serotype 19A in the Netherlands and lower rates of serogroup 15A/15B/15C/15F and serotype 21 in England. This finding is likely attributable to differences in national vaccination program, with the vaccine used to immunize young children in the Netherlands not targeting serotypes 3, 6A, and 19A.

Age-related declines in pneumococcal colonization of the nasopharynx were accompanied by declines in *M. catarrhalis* and *H. influenzae* and a simultaneous increase in *S. aureus* colonization rates. Numerous other studies have reported age-related differences in nasopharyngeal colonization by pneumococcus [36–38] and of other bacterial species [12–14, 39]. Although studies typically attribute age-specific colonization rates to differences in social interactions or immunological status, such differences cannot explain increased dissimilarity in pneumococcal serotype composition with age among paired nasopharyngeal and saliva samples.

It is probable that age-related shifts in bacterial colonization of the nasopharynx are linked to postnatal histological differentiation of the nasopharyngeal mucosa. In neonates and infants, the nasopharynx consists almost solely of ciliated pseudostratified columnar epithelium [40–44]. However, during postnatal development, the pseudostratified columnar epithelium is gradually replaced with intermediate epithelium and non-keratinizing stratified squamous epithelium [40–43, 45, 46]. Upon reaching adulthood, squamous epithelium may comprise ~60% of the anterior and 80% of the posterior nasopharyngeal wall [40, 43]. This age-related differentiation of the nasopharyngeal epithelium may be accompanied by an overall decline in goblet cell densities [43, 47, 48]. Consequently, adults may exhibit diminished production of mucin when compared with young children [41, 47]. Importantly, utilization of mucin-derived carbohydrates, such as galactose, likely enables pneumococcus to colonize the nasopharynx [35, 49, 50].

**Figure 3:**
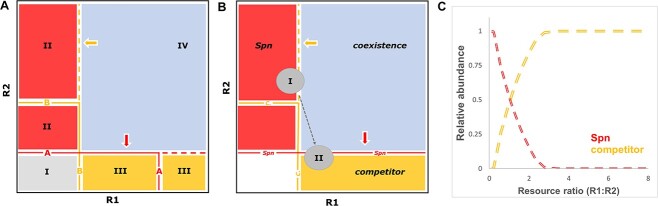
Graphical model of hypothetical age-related resource gradient and consumer-resource dynamics of pneumococcus in the nasopharynx; (A) in the graphical model, the equilibrium outcomes of resource competition between two species (A and B) consuming two substitutable resources (R1 and R2) are illustrated; the solid lines represent the zero-net growth isoclines (ZNGIs), which correspond to the amounts of resources a habitat must possess for the population growth rates of the species to equal population mortality rates, and the vectors indicate the slope of the resource consumption rate; the ZNGIs (requirement niche) and impact vectors (impact niche; indicated by arrows) define the ecological niche of a species [2]; habitats with supply points that are located above the ZNGI of a species will produce positive growth rates for that species; for coexistence to occur between species competing for two resources, the species are expected to display trade-offs in resource consumption as indicated by the intersection of ZNGIs; the ZNGIs take diauxic growth of bacterial species in consideration [5, 6, 53], and the species with the lowest ZNGI for a particular resource is a superior competitor for that resource as it can deplete resource levels in an habitat below the ZNGI of its competitors; the equilibrium outcomes of resource competition are labelled with I, II, III, and IV; in habitats corresponding to resource supply points of Region I, resource levels are insufficient for either species to maintain non-negative population growth rates; in habitats corresponding to resource supply points of Region II, resource ratios favour species A and species B will be competitively excluded; the opposite competitive outcome is observed for resource supply points corresponding to Region III; in Region IV, resource supply points favour both species, and species A and B can coexist when there is no strong interference competition between both species; in case of strong interference competition, priority effects may determine which species will become dominant; (B) a hypothetical scenario of resource competition between *S. pneumoniae* and other bacterial species colonizing the nasopharynx; the solid lines represent ZNGIs for pneumococcus and it’s competitor; here, *S. pneumoniae* (“Spn”) only consumes R2, whereas the competing species (“C.”) can consume both R1 and R2; in this scenario, age-related differences in resource levels and resource ratios are indicated as a declining resource gradient (dashed line) from supply Point I to II, representing the infant nasopharynx and adult nasopharynx, respectively; this resource gradient depicts a decline in R2 with increasing age during early life, R2 represents a mucin-derived resource (i.e. galactose); R1 represents a resource not derived from mucin and which displays an increase in its availability along the resource gradient; in adults (II), mucin-derived resources are insufficient to sustain positive *S. pneumoniae* population growth rates and a competing species, e.g. *S. aureus,* may exhibit increased competitive dominance in the nasopharynx; alternatively, resource levels may largely remain above the ZNGIs of *S. pneumoniae,* but resource ratios strongly favour a superior competitor (scenario not shown); (C) the decline in resource levels and resource ratios along the resource gradient (see B) are associated with decreasing abundances in pneumococcus due to shifts in competitive dominance.

We hypothesize that declines in mucin-derived resources due to postnatal histological changes of the nasopharynx may impact nasopharyngeal colonization rates of pneumococcus and drive age-related differences in the microbiota composition of the nasopharynx. Here, we propose a conceptual model based on ecological consumer-resource models to characterize the age-related dynamics of pneumococcal carriage in the nasopharynx. These mechanistic models have been used to study characteristics of microbial communities [51, 52] and presume that species possess competitive trade-offs (Fig. 3A). According to this framework, competitive dominance depends on resource availability within a habitat and resource utilization trade-offs [2, 3]. In other words, trade-offs are important drivers of the composition of microbial communities, and trade-offs related to the utilization of limiting resources are of particular importance. As such, resource availability within a habitat can play an important role in shaping the composition of the microbial community. Consequently, the transformation of the nasopharyngeal epithelium within the first decade of life may produce an age-related resource gradient, which negatively impacts the competitive ability of pneumococcus to colonize the nasopharynx (Fig. 3B and C), resulting in diminishing nasopharyngeal colonization rates and abundances.

Applying the concepts of consumer-resource theory to the context of pneumococcal colonization suggests that the environmental conditions of the nasopharynx in young children may considerably favour pneumococcal colonization as the nasopharynx is relatively rich in ciliated epithelium and mucin-derived resources. Moreover, viral respiratory tract infections, which are more frequent among young children [54], may facilitate pneumococcal colonization through induction of mucin production by goblet cells [55]. As such, in the nasopharynx of young children, pneumococcus may outcompete bacterial species that less efficiently utilize mucin-derived resources or are more prone to mucociliary clearance. Consequently, diminishing abundances of mucin-derived resources in the developing nasopharynx may shift resource ratios in favour of bacterial species that are not largely dependent on mucin. For example, *S. aureus,* which primary reservoir is considered to be the anterior nares [56], displays higher nasopharyngeal colonization rates in older children and adults, and possibly increased competitive dominance, when the nasopharyngeal environment may become relatively poor in mucin-derived resources [39]. Accordingly, the ecological niche of pneumococcus, represented by a requirement component and an impact component [2, 3], is largely restricted to the oral cavity in adults, as the environmental conditions of the adult nasopharynx may inadequately satisfy the requirement niche of pneumococcus and increasingly resemble the environmental conditions of the anterior nares. Likewise, in nasopharyngeal samples from children who were positive for *S. aureus* by culture, the nasopharyngeal overall bacterial abundances were significantly decreased when compared with samples negative for *S. aureus* by culture. These findings mirror observations described by Stearns *et al.* [12] and may represent the nasopharyngeal environment in nutrient-rich (e.g. relatively high availability of mucin-derived resources) conditions with increased colonization by *S. pneumoniae*, *H. influenzae*, and *M. catarrhalis* and high overall bacterial abundances and in nutrient-poor conditions with increased colonization by *S. aureus*.

High interindividual variation in age-related effects on nasopharyngeal histology and viral respiratory tract infections may explain pneumococcal colonization in the nasopharynx of some adults. Moreover, metacommunity frameworks indicate that if dispersal rates are high, sink-source dynamics (mass effects) may occur during which competitively inferior species can avoid competitive exclusion [57]. Consequently, it indicates that adults with high exposure to pneumococcal shedding may exhibit transient pneumococcal colonization of the nasopharynx.

This study has several limitations. First, comparisons of pneumococcal serotype composition of respiratory samples with Bray–Curtis dissimilarity were limited to qPCR-targeted serotypes. Therefore, certain serotypes or serogroups in circulation at that time in England or the Netherlands may have been missed. Second, culture-enriched saliva samples were not processed in the cohort from England, for this reason analysis of data from England is limited to nasopharyngeal samples. Third, nasopharyngeal culture data included only a limited number of bacterial species, many of which are bacterial pathogens, and no microbiota analysis was conducted to expand the analysis on presumed age-related differences of nasopharyngeal samples. An age-related gradient associated with immune development may also impact bacterial colonization in the upper airways, where immune-mediated apparent competition occurs [58, 59]. This immune-mediated competition likely operates in conjunction with consumer-resource dynamics. However, immune status is unlikely to contribute strongly to age-related colonization differences in different anatomical sites of the upper airways (oral site versus nasopharynx within the same individual). Finally, we were not able to test the framework we have proposed here as we have not quantified and characterized respiratory samples for (host-derived) resources.

In conclusion, findings from this study suggest that the environmental conditions of the nasopharynx utilized by pneumococcus and other bacteria are likely to be dynamic and exhibit age-related differences. Such age-related changes of the nasopharynx can result in host-driven ecological succession of bacterial species colonizing the nasopharynx and lead to competitive exclusion of pneumococcus from the nasopharynx. It also may explain poor performance of nasopharyngeal samples for surveillance of pneumococcal carriage among adults. Our observations indicate that pneumococci detected using saliva samples may more accurately resemble the epidemiology of pneumococcal carriage among adults.

## Supplementary Material

fig_S1_20231215_ycae002

fig_S2_20231217_2_ycae002

fig_S3_20231217_2_ycae002

table_S1_revised_ycae002

table_S2_ycae002

table_S3_ycae002

table_S4_ycae002

table_S5_ycae002

table_S6_ycae002

table_S7_ycae002

table_S8_revised_ycae002

table_S9_revised_ycae002

table_S10_revised_ycae002

table_S11_revised_ycae002

Supplementary_methods_ycae002
