# Supplementary figures and images for: Impact of age on pneumococcal colonization of the nasopharynx and oral cavity: an ecological perspective
Source: ISME Commun. 2024 Jan 12;4(1):ycae002. doi: 10.1093/ismeco/ycae002 (PMC10881297; doi:10.1093/ismeco/ycae002)

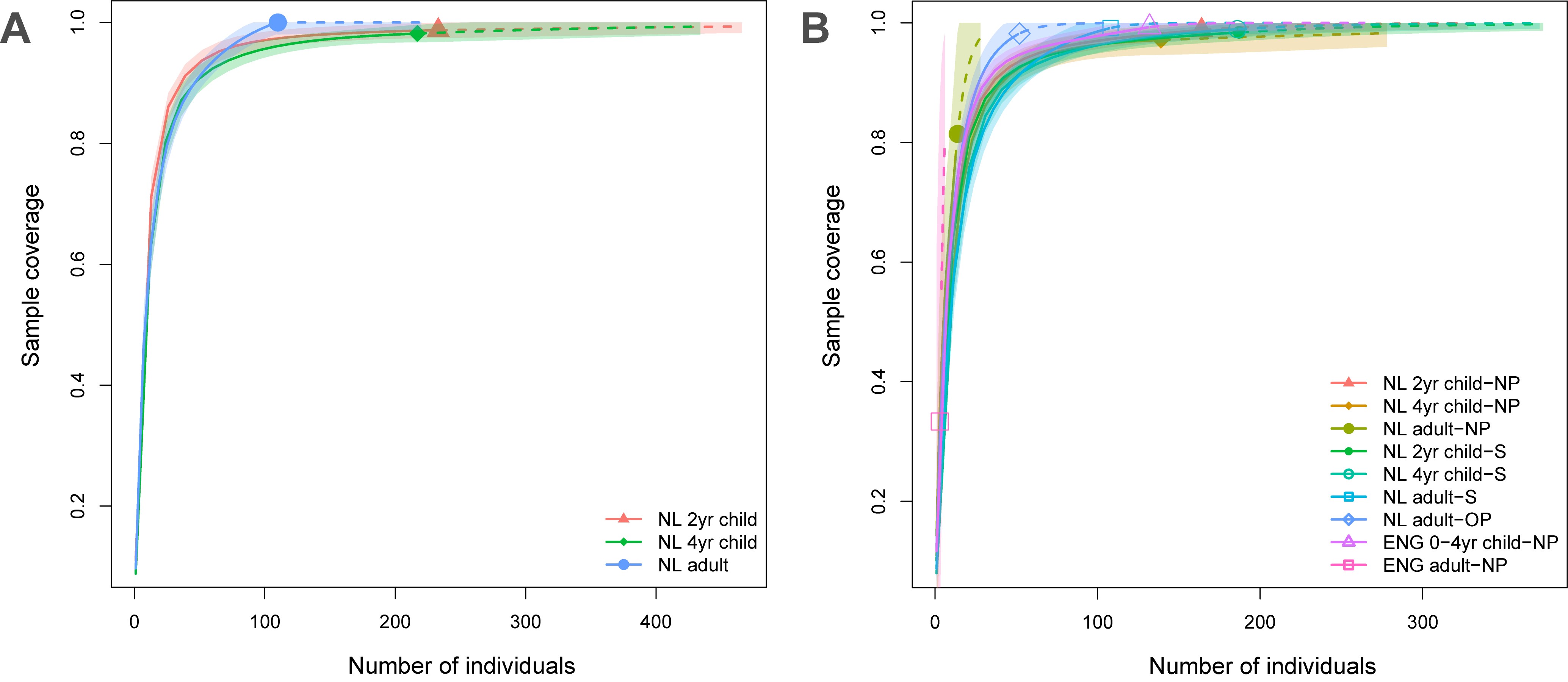

Supplement: fig_S1_20231215_ycae002 [file fig_s1_20231215_ycae002.jpeg]

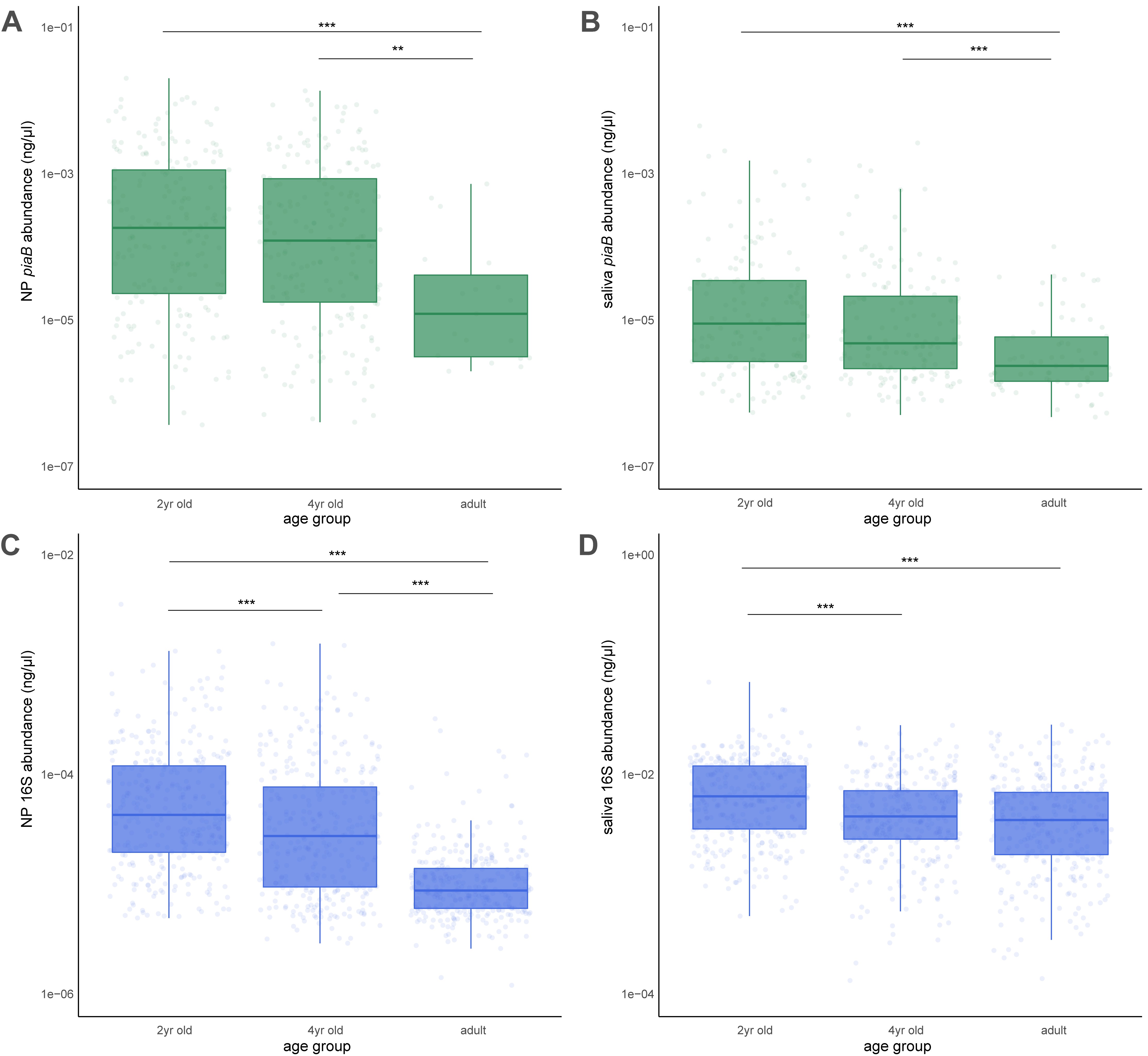

Supplement: fig_S2_20231217_2_ycae002 [file fig_s2_20231217_2_ycae002.jpeg]

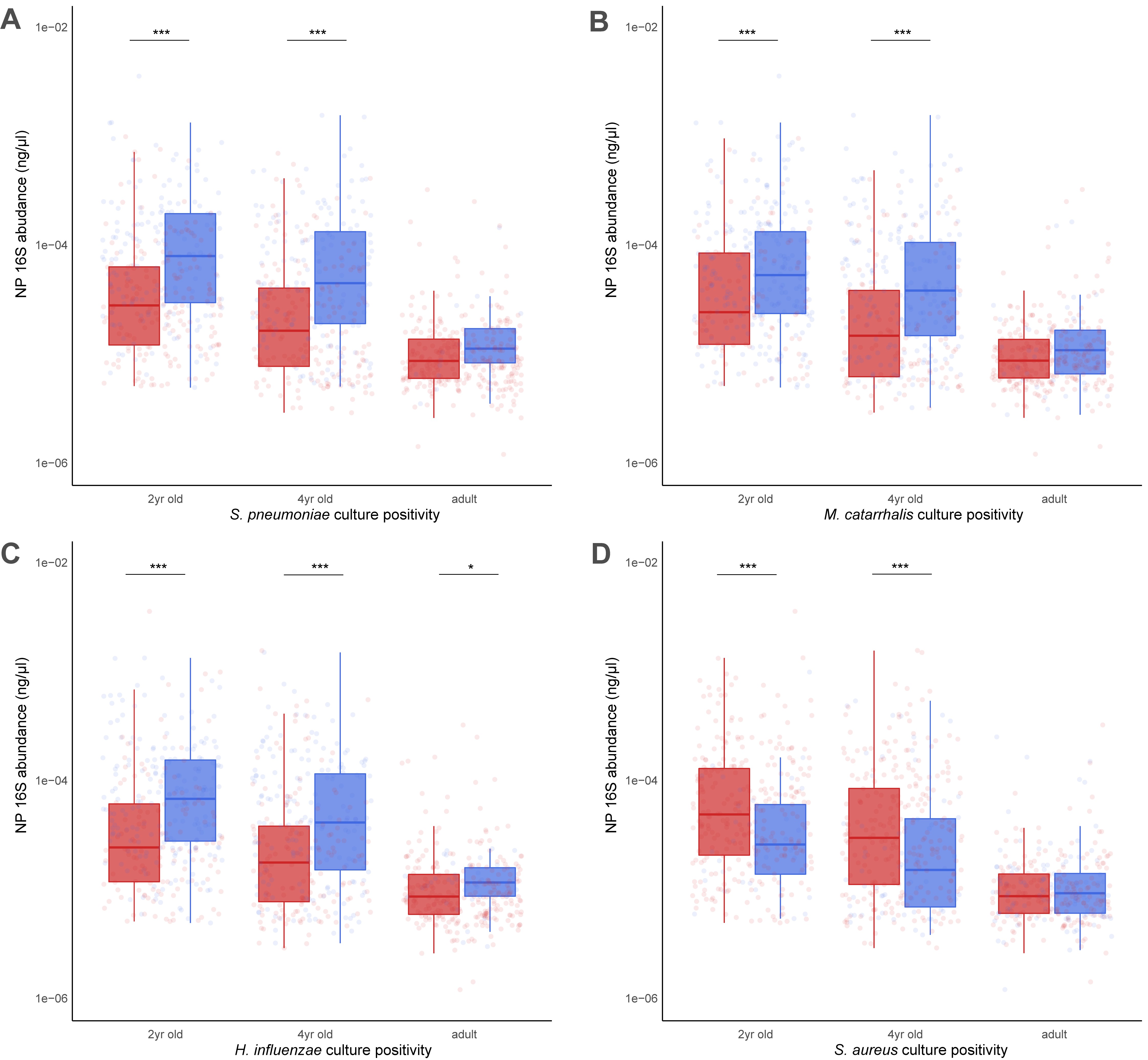

Supplement: fig_S3_20231217_2_ycae002 [file fig_s3_20231217_2_ycae002.jpeg]
