# Supplementary material for: Impact of age on pneumococcal colonization of the nasopharynx and oral cavity: an ecological perspective
Source: ISME Commun. 2024 Jan 12;4(1):ycae002. doi: 10.1093/ismeco/ycae002 (PMC10881297; doi:10.1093/ismeco/ycae002)
Supplement: table_S1_revised_ycae002 [file table_s1_revised_ycae002.docx]

**Table S1: C_q_ thresholds derived with receiver operating characteristics curve analysis and based on amplifying slopes as criterion.**

| Sample | Study | Criterion | Optimal threshold  *piaB* (95% CI) | Youden index  *piaB* | Sensitivity  *piaB* | Specificity  *piaB* | Optimal threshold  *lytA* (95% CI) | Youden index  *lytA* | Sensitivity  *lytA* | Specificity  *lytA* | Reference |
| --- | --- | --- | --- | --- | --- | --- | --- | --- | --- | --- | --- |
| NP | NL | ‘amplification slope’ | 35.84 (29.65-36.61) | 0.99 | 1 | 0.99 | 32.3 (29.29-36.22) | 0.97 | 1 | 0.97 | This study |
| NP | ENG |  | 34.19 (27.29-35.71) | 0.99 | 0.99 | 1 | 33.74 (27-95-37.55) | 0.98 | 0.99 | 0.98 | This study |
| OP^*^ | NL |  | 37.53 (35.28-37.77) | 0.96 | 0.98 | 0.98 | 34.15 (32.22-37.63) | 0.86 | 0.91 | 0.96 | This study |
| saliva | NL |  | 38.03 (36.79-39.49) | 0.92 | 0.98 | 0.94 | 36.85 (35.78-37.52) | 0.83 | 0.97 | 0.85 | (10) |

^*^: OP samples were only collected from adults and not from children. NP: nasopharyngeal, OP: oropharyngeal, NL: cohort from the Netherlands, ENG: cohort from England.
