# Supplementary material for: Impact of age on pneumococcal colonization of the nasopharynx and oral cavity: an ecological perspective
Source: ISME Commun. 2024 Jan 12;4(1):ycae002. doi: 10.1093/ismeco/ycae002 (PMC10881297; doi:10.1093/ismeco/ycae002)
Supplement: table_S3_ycae002 [file table_s3_ycae002.docx]

**Table S3: Serotypes detected in 4-year-old children (n=326) in NL study**

| serotype/ | nasopharyngeal sample | | |  | saliva |  | overall | | |
| --- | --- | --- | --- | --- | --- | --- | --- | --- | --- |
| /serogroup | culture | qPCR | overall |  | qPCR |  | culture | qPCR | overall |
| 1 | 0 (0) | 0 (0) | 0 (0) |  | 1 (0.3) |  | 0 (0) | 1 (0.3) | 1 (0.3) |
| 3 | 6 (1.8) | 14 (4.3) | 14 (4.3) |  | 14 (4.3) |  | 6 (1.8) | 22 (6.7) | 22 (6.7) |
| 4 |  | NR |  |  | NR |  |  | NR |  |
| 5 |  | NR |  |  | NR |  |  | NR |  |
| 6A/B/C/D | 17 (5.2) | 19 (5.8) | 20 (6.1) |  | 17 (5.2) |  | 17 (5.2) | 25 (7.7) | 26 (8) |
| 7A/F | 0 (0) | 0 (0) | 0 (0) |  | 2 (0.6) |  | 0 (0) | 2 (0.6) | 2 (0.6) |
| 7C | 0 (0) | ND | 0 (0) |  | ND |  | 0 (0) | ND | 0 (0) |
| 8 | 2 (0.6) | 3 (0.9) | 3 (0.9) |  | 5 (1.5) |  | 2 (0.6) | 5 (1.5) | 5 (1.5) |
| 9A/L/N/V | 2 (0.6) | 2 (0.6) | 2 (0.6) |  | NR |  | 2 (0.6) | 2 (0.6) | 2 (0.6) |
| 10A/B | 4 (1.2) | 6 (1.8) | 6 (1.8) |  | 7 (2.1) |  | 4 (1.2) | 10 (3.1) | 10 (3.1) |
| 11A/D | 11 (3.4) | 15 (4.6) | 15 (4.6) |  | 17 (5.2) |  | 11 (3.4) | 20 (6.1) | 20 (6.1) |
| 12A/B/F | 0 (0) | 0 (0) | 0 (0) |  | NR |  | 0 (0) | 0 (0) | 0 (0) |
| 14 | 0 (0) | 1 (0.3) | 1 (0.3) |  | 1 (0.3) |  | 0 (0) | 1 (0.3) | 1 (0.3) |
| 15A/B/C/F | 9 (2.8) | 13 (4) | 13 (4) |  | 19 (5.8) |  | 9 (2.8) | 26 (8) | 26 (8) |
| 16F | 5 (1.5) | 7 (2.1) | 7 (2.1) |  | 11 (3.4) |  | 5 (1.5) | 13 (4) | 13 (4) |
| 17F | 3 (0.9) | NR | 3 (0.9) |  | NR |  | 3 (0.9) | NR | 3 (0.9) |
| 18A/B/C/F | 0 (0) | 0 (0) | 0 (0) |  | 0 (0) |  | 0 (0) | 0 (0) | 0 (0) |
| 19A | 16 (4.9) | 21 (6.4) | 21 (6.4) |  | 31 (9.5) |  | 16 (4.9) | 37 (11.3) | 37 (11.3) |
| 19F | 3 (0.9) | 3 (0.9) | 3 (0.9) |  | 6 (1.8) |  | 3 (0.9) | 6 (1.8) | 6 (1.8) |
| 20 | 1 (0.3) | 1 (0.3) | 1 (0.3) |  | 0 (0) |  | 1 (0.3) | 1 (0.3) | 1 (0.3) |
| 21 | 4 (1.2) | 5 (1.5) | 5 (1.5) |  | 12 (3.7) |  | 4 (1.2) | 14 (4.3) | 14 (4.3) |
| 22A/F | 2 (0.6) | 2 (0.6) | 2 (0.6) |  | 2 (0.6) |  | 2 (0.6) | 3 (0.9) | 3 (0.9) |
| 23A | 6 (1.8) | 8 (2.5) | 8 (2.5) |  | 17 (5.2) |  | 6 (1.8) | 19 (5.8) | 19 (5.8) |
| 23B | 16 (4.9) | 18 (5.5) | 19 (5.8) |  | 20 (6.1) |  | 16 (4.9) | 26 (8) | 27 (8.3) |
| 23F | 0 (0) | 0 (0) | 0 (0) |  | 2 (0.6) |  | 0 (0) | 2 (0.6) | 2 (0.6) |
| 24(F) | 6 (1.8) | ND | 6 (1.8) |  | ND |  | 6 (1.8) | ND | 6 (1.8) |
| 31 | 0 (0) | ND | 0 (0) |  | ND |  | 0 (0) | ND | 0 (0) |
| 33A/F/37 | 5 (1.5) | 6 (1.8) | 6 (1.8) |  | 4 (1.2) |  | 5 (1.5) | 7 (2.1) | 7 (2.1) |
| 34 | 1 (0.3) | 1 (0.3) | 1 (0.3) |  | 3 (0.9) |  | 1 (0.3) | 3 (0.9) | 3 (0.9) |
| 35B/C | 4 (1.2) | 5 (1.5) | 5 (1.5) |  | NR |  | 4 (1.2) | 5 (1.5) | 5 (1.5) |
| 35F | 8 (2.5) | ND | 8 (2.5) |  | ND |  | 8 (2.5) | ND | 8 (2.5) |
| 38 | 0 (0) | 1 (0.3) | 1 (0.3) |  | 4 (1.2) |  | 0 (0) | 5 (1.5) | 5 (1.5) |

*NR: not reliable by qPCR, ND: not determined,* NL: cohort from the Netherlands.
