# Supplementary material for: Impact of age on pneumococcal colonization of the nasopharynx and oral cavity: an ecological perspective
Source: ISME Commun. 2024 Jan 12;4(1):ycae002. doi: 10.1093/ismeco/ycae002 (PMC10881297; doi:10.1093/ismeco/ycae002)
Supplement: table_S5_ycae002 [file table_s5_ycae002.docx]

**Table S5: Serotypes detected in children younger than 5 years old (n=293) in ENG study**

| serotype/ | nasopharyngeal sample | | |  |
| --- | --- | --- | --- | --- |
| /serogroup | culture | qPCR | overall |  |
| 1 | 0 (0) | 0 (0) | 0 (0) | |
| 3 | 0 (0) | 2 (0.7) | 2 (0.7) | |
| 4 | 0 | NR | 0 | |
| 5 | 0 | NR | 0 | |
| 6A/B/C/D | 1 (0.3) | 2 (0.7) | 2 (0.7) | |
| 7A/F | 0 (0) | 0 (0) | 0 (0) | |
| 7C | 3 (1.1) | ND | 3 (1.1) | |
| 8 | 0 (0) | 0 (0) | 0 (0) | |
| 9A/L/N/V | 3 (1) | 6 (2) | 6 (2) | |
| 10A/B | 7 (2.4) | 10 (3.4) | 11 (3.8) | |
| 11A/D | 15 (5.1) | 17 (5.8) | 20 (6.8) | |
| 12A/B/F | 0 (0) | 1 (0.3) | 1 (0.3) | |
| 14 | 0 (0) | 0 (0) | 0 (0) | |
| 15A/B/C/F | 22 (7.7) | 30 (10.2) | 35 (11.9) | |
| 16F | 9 (3.1) | 12 (4.1) | 12 (4.1) | |
| 17F | 4 (1.4) | NR | 4 (1.4) | |
| 18A/B/C/F | 0 (0) | 0 (0) | 0 (0) | |
| 19A | 2 (0.7) | 2 (0) | 2 (0.7) | |
| 19F | 0 (0) | 0 (0) | 0 (0) | |
| 20 | 0 (0) | 0 (0) | 0 (0) | |
| 21 | 9 (3.1) | 14 (4.8) | 15 (5.1) | |
| 22A/F | 5 (1.7) | 6 (2) | 6 (2) | |
| 23A | 9 (3.1) | 9 (3.1) | 9 (3.1) | |
| 23B | 15 (5.1) | 19 (6.5) | 21 (7.2) | |
| 23F | 0 (0) | 0 (0) | 0 (0) | |
| 24(F) | 3 (1.1) | ND | 3 (1.1) | |
| 31 | 7 (2.5) | ND | 7 (2.5) | |
| 33A/F/37 | 5 (1.7) | 6 (2) | 9 (3.1) | |
| 34 | 1 (0.3) | 1 (0.3) | 1 (0.3) | |
| 35B/C | 4 (1.4) | 7 (2.4) | 7 (2.4) | |
| 35F | 3 (1.1) | ND | 3 (1.1) | |
| 38 | 2 (0.7) | 2 (0.7) | 2 (0.7) | |

*NR: not reliable by qPCR, ND: not determined,* ENG: cohort from England.
