# Supplementary material for: Impact of age on pneumococcal colonization of the nasopharynx and oral cavity: an ecological perspective
Source: ISME Commun. 2024 Jan 12;4(1):ycae002. doi: 10.1093/ismeco/ycae002 (PMC10881297; doi:10.1093/ismeco/ycae002)
Supplement: table_S6_ycae002 [file table_s6_ycae002.docx]

**Table S6: Serotypes detected in adults older than 20 years old (n=284) in ENG study**

| serotype/ | nasopharyngeal sample | | |
| --- | --- | --- | --- |
| /serogroup | culture | qPCR | overall |
| 1 | 0 (0) | 0 (0) | 0 (0) |
| 3 | 0 (0) | 0 (0) | 0 (0) |
| 4 | 0 | NR | 0 |
| 5 | 0 | NR | 0 |
| 6A/B/C/D | 0 (0) | 0 (0) | 0 (0) |
| 7A/F | 0 (0) | 0 (0) | 0 (0) |
| 7C | 0 (0) | ND | 0 (0) |
| 8 | 0 (0) | 0 (0) | 0 (0) |
| 9A/L/N/V | 1 (0.4) | 1 (0.4) | 1 (0.4) |
| 10A/B | 1 (0.4) | 0 (0) | 1 (0.4) |
| 11A/D | 0 (0) | 0 (0) | 0 (0) |
| 12A/B/F | 0 (0) | 0 (0) | 0 (0) |
| 14 | 0 (0) | 0 (0) | 0 (0) |
| 15A/B/C/F | 1 (0.4) | 1 (0.4) | 1 (0.4) |
| 16F | 0 (0) | 0 (0) | 0 (0) |
| 17F | 1 (0.4) | NR | 1 (0.4) |
| 18A/B/C/F | 0 (0) | 0 (0) | 0 (0) |
| 19A | 0 (0) | 0 (0) | 0 (0) |
| 19F | 0 (0) | 0 (0) | 0 (0) |
| 20 | 0 (0) | 0 (0) | 0 (0) |
| 21 | 0 (0) | 0 (0) | 0 (0) |
| 22A/F | 0 (0) | 0 (0) | 0 (0) |
| 23A | 1 (0.4) | 1 (0.4) | 1 (0.4) |
| 23B | 0 (0) | 0 (0) | 0 (0) |
| 23F | 0 (0) | 0 (0) | 0 (0) |
| 24F | 0 (0) | ND | 0 (0) |
| 31 | 1 (0.4) | ND | 1 (0.4) |
| 33A/F/37 | 1 (0.4) | 1 (0.4) | 1 (0.4) |
| 34 | 0 (0) | 0 (0) | 0 (0) |
| 35B/C | 1 (0.4) | 1 (0.4) | 1 (0.4) |
| 35F | 0 (0) | ND | 0 (0) |
| 38 | 0 (0) | 0 (0) | 0 (0) |

*NR: not reliable by qPCR, ND: not determined,* ENG: cohort from England.
