# Supplementary material for: Impact of age on pneumococcal colonization of the nasopharynx and oral cavity: an ecological perspective
Source: ISME Commun. 2024 Jan 12;4(1):ycae002. doi: 10.1093/ismeco/ycae002 (PMC10881297; doi:10.1093/ismeco/ycae002)
Supplement: table_S7_ycae002 [file table_s7_ycae002.docx]

**Table S7 : Serotype (alpha) diversity per specimen-type-age-group combination**

| Group | Sample | Richness  (n serotypes detected) | Shannon index |
| --- | --- | --- | --- |
| 2-year old NL children | NP | 164 | 2.37 |
|  | saliva | 187 | 2.43 |
| 4-year-old NL children | NP | 139 | 2.53 |
|  | saliva | 186 | 2.60 |
| 0-4-year-old ENG children | NP | 132 | 2.29 |
| NL adults | NP | 14 | 1.77 |
|  | OP | 52 | 2.18 |
|  | saliva | 108 | 2.59 |
| ENG adults | NP | 3 | 1.10 |

NL: cohort from the Netherlands, ENG: cohort from England.
