# Supplementary material for: Impact of age on pneumococcal colonization of the nasopharynx and oral cavity: an ecological perspective
Source: ISME Commun. 2024 Jan 12;4(1):ycae002. doi: 10.1093/ismeco/ycae002 (PMC10881297; doi:10.1093/ismeco/ycae002)
Supplement: table_S8_revised_ycae002 [file table_s8_revised_ycae002.docx]

**Table S8: Nasopharyngeal culture rates for *Streptococcus pneumoniae, Moraxella catarrhalis, Haemophilus influenzae, Staphylococcus aureus* and β-haemolytic Streptococci cultured in the Netherlands from 2-year-old children** (n=327)**, 4-year-old children** (n=326) **and parents of 2-year-old children** (n=318).

| Culture | 2-year-old  children  n (%) | 4-year-old  children  n (%) | adults  n (%) |
| --- | --- | --- | --- |
| *S. pneumoniae* | 158 (48.3) | 148 (45.4) | 19 (6) |
| *M. catarrhalis* | 238 (72.8) | 206 (63.2) | 44 (13.8) |
| *H. influenzae* | 197 (60.2) | 177 (54.3) | 35 (11) |
| *S. aureus* | 36 (11) | 60 (18.4) | 109 (34.3) |
| β-haemolytic Streptococci* | 7 (1.1) | 18 (5.5) | 3 (0.9) |

*: This included *Streptococcus pyogenes, Streptococcus dysgalactiae and Streptococcus anginosus. Other β-haemolytic streptococcal species were not cultured.*
