# Supplementary material for: Impact of age on pneumococcal colonization of the nasopharynx and oral cavity: an ecological perspective
Source: ISME Commun. 2024 Jan 12;4(1):ycae002. doi: 10.1093/ismeco/ycae002 (PMC10881297; doi:10.1093/ismeco/ycae002)
Supplement: table_S9_revised_ycae002 [file table_s9_revised_ycae002.docx]

**Table S9: Associations between bacteria isolated from nasopharyngeal cultures from NL 2-year-old children (n=327)**

| species x | species y | number of  co-occurences | probability  co-occurences | expected  co-occurences | negative  association (*p*)^†^ | positive  association (*p*) ^†^ |
| --- | --- | --- | --- | --- | --- | --- |
| *Streptococcus pneumoniae* | *Haemophilus influenzae* | 116 | 0.291 | 95.2 | 1.00000 | 0.00000 |
| *Streptococcus pneumoniae* | *Moraxella catarrhalis* | 132 | 0.352 | 115 | 0.99999 | 0.00002 |
| *Streptococcus pneumoniae* | *Staphylococcus aureus* | 11 | 0.053 | 17.4 | 0.01774 | 0.99323 |
| *Haemophilus influenzae* | *Moraxella catarrhalis* | 159 | 0.438 | 143.4 | 0.99998 | 0.00007 |
| *Haemophilus influenzae* | *Staphylococcus aureus* | 13 | 0.066 | 21.7 | 0.00172 | 0.99949 |
| *Moraxella catarrhalis* | *Staphylococcus aureus* | 19 | 0.08 | 26.2 | 0.00521 | 0.99829 |

Only significant associations are included in the table. NL: cohort from the Netherlands. ^†^: Negative and positive associations are indicated by pairwise probabilities, probabilities below <0.05 are regarded as significant.
