# Supplementary material for: Impact of age on pneumococcal colonization of the nasopharynx and oral cavity: an ecological perspective
Source: ISME Commun. 2024 Jan 12;4(1):ycae002. doi: 10.1093/ismeco/ycae002 (PMC10881297; doi:10.1093/ismeco/ycae002)
Supplement: table_S10_revised_ycae002 [file table_s10_revised_ycae002.docx]

**Table S10: Associations between bacteria isolated from nasopharyngeal cultures from NL 4-year-old children (n=326)**

| species x | species y | number of  co-occurences | probability  co-occurences | expected  co-occurences | negative  association (*p*) ^†^ | positive  association (*p*) ^†^ |
| --- | --- | --- | --- | --- | --- | --- |
| *Streptococcus pneumoniae* | *Haemophilus influenzae* | 96 | 0.245 | 80.3 | 0.99985 | 0.00034 |
| *Streptococcus pneumoniae* | *Moraxella catarrhalis* | 105 | 0.286 | 93.9 | 0.99648 | 0.00691 |
| *Streptococcus pneumoniae* | *Staphylococcus aureus* | 21 | 0.084 | 27.5 | 0.04208 | 0.97821 |
| *Haemophilus influenzae* | *Moraxella catarrhalis* | 125 | 0.344 | 112.9 | 0.99816 | 0.00374 |
| *Moraxella catarrhalis* | *Staphylococcus aureus* | 26 | 0.118 | 39.7 | 0.00020 | 0.99993 |

Only significant associations are included in the table, NL: cohort from the Netherlands. ^†^: Negative and positive associations are indicated by pairwise probabilities, probabilities below <0.05 are regarded as significant.
