# Supplementary material for: Impact of age on pneumococcal colonization of the nasopharynx and oral cavity: an ecological perspective
Source: ISME Commun. 2024 Jan 12;4(1):ycae002. doi: 10.1093/ismeco/ycae002 (PMC10881297; doi:10.1093/ismeco/ycae002)
Supplement: table_S11_revised_ycae002 [file table_s11_revised_ycae002.docx]

**Table S11: Associations between bacteria isolated from nasopharyngeal cultures from NL adults (n=318)**

| species x | species y | number of  co-occurences | probability  co-occurences | expected  co-occurences | negative  association (*p*) ^†^ | positive  association (*p*) ^†^ |
| --- | --- | --- | --- | --- | --- | --- |
| *Haemophilus influenzae* | *Moraxella catarrhalis* | 10 | 0.015 | 4.8 | 0.99683 | 0.01101 |

Only significant associations are included in the table, NL: cohort from the Netherlands. ^†^: Negative and positive associations are indicated by pairwise probabilities, probabilities below <0.05 are regarded as significant.
