# Supplementary material for: Impact of age on pneumococcal colonization of the nasopharynx and oral cavity: an ecological perspective
Source: ISME Commun. 2024 Jan 12;4(1):ycae002. doi: 10.1093/ismeco/ycae002 (PMC10881297; doi:10.1093/ismeco/ycae002)
Supplement: Supplementary_methods_ycae002 [file supplementary_methods_ycae002.docx]

**Supplementary methods**

*Nasopharyngeal and oropharyngeal swab collection*

Immediately after the sampling procedure, nasopharyngeal or oropharyngeal swabs in the Netherlands were placed in liquid Amies transport medium (ESwab 482C, Copan, Brescia, Italy) and within 8 h transported to the diagnostic laboratory. In England nasopharyngeal swabs were placed in skim-milk, tryptone, glucose and glycerol (STGG) broth and delivered to a diagnostic laboratory within 48 h.
